# Turning Points In My Medical Career

**DOI:** 10.4103/0973-1229.27612

**Published:** 2006

**Authors:** Sunil Pandya

**Affiliations:** * *Department of Neurosurgery, Jaslok Hospital & Research Centre, Dr. G. V. Deshmukh Marg, Mumbai 400020, India Email:*shunil@vsnl.com

**Keywords:** Teachers, Medical Ethics

## Abstract

I have reviewed briefly persons who have influenced me during my years as a student of medicine and to date. I have been blessed in my teachers and owe everything I am to them. The chief lessons they taught me were integrity, sincerity, the need to keep learning and practice ethically keeping the welfare of the patient in mind all the time. Above all, they taught me to observe the Golden Rule**.

## Introduction

I have learnt so much from so many. I continue to learn, especially from my colleagues, juniors, patients and their families and from books and journals. It is difficult to identify ‘turning points’ as my learning forms a continuum. I find it easier to describe what I have learnt from some of my teachers.

## Why I Became A Medical Doctor

I am unsure about the origins of my desire to choose medicine as a vocation. In retrospect, there must have been a combination of factors.

I had closely watched my uncle, who was a family physician in Lonavala, Maharashtra, and witnessed the trust and respect he inspired in his patients and their families. Dr. C. S. Thakkar, our family physician in Santa Cruz, and his nephew, also a doctor (whom we affectionately addressed as Champukaka) have left indelible impressions on my mind.

My mother had instilled into my brother and me the values of honesty, humanity and humility. Our family was of modest means and at times had difficulty meeting recurring expenses. My aunt in Juhu and uncle in Surat often helped us. Complementing their efforts was the generosity of our landlord who did not say a word even when we had not paid him rent over a year! Such experiences sensitised me to the plight of the less fortunate and inspired a desire to emulate those who had helped us in our need. Medicine appealed to me as it provided an opportunity to exercise qualities of the head and heart.

As I entered Ismail Yusuf College in Jogeshwari after passing the Secondary School Certificate examination, I chose to study science and preferred biology to engineering. The decision was partly based on my lack of enthusiasm for mathematics. I did not find the dissection of the cockroach, frog and lobster repulsive. Around this time, I also became aware of the life and work of Albert Schweitzer. I was to learn much more about him after I had joined medical college and will discuss his influence later.

I approached medicine as a calling or vocation. I was aware that I had to earn my livelihood by my practice but cannot recall an urge to reach the then hallowed status of a ‘*lakhpati*’*.

## The Enforced Sabbatical In 1956

Due to an unusual set of circumstances, I had passed my intermediate examination in science at the age of 16 years. The minimum age for entering medical college was 17 years. I enjoyed a year-long holiday. My fondness for books led to membership of the library of the United States Information Service (as it was called then). I preferred this library to that of the British Council as membership was free of cost and as it was near Churchgate railway station in Mumbai. I benefited greatly from it.

Let me give you just one example. I chanced upon the biography of George Washington Carver. I learnt of this Negro scientist (the term ‘Negro’ was acceptable then and had not yet been replaced by ‘African American’), who worked in Tuskegee and, by his studies, revolutionised the agricultural economy of the poor in the southern American states by showing that 300 products could be derived from the humble peanut. By 1938, peanuts had become a $200 million industry and the chief product of Alabama. Carver also demonstrated that 100 different products could be derived from the sweet potato. Carver did not patent the many discoveries he made while at Tuskegee, saying ‘God gave them to me, how can I sell them to someone else?’ (Wilhelm Roentgen and Louis Pasteur also expressed this sentiment. They, too, chose not to profit from their discoveries.). Carver experienced awe in his encounter with the natural world.

Mahatma Gandhi approached him for advice on nutritious diet for the poor.

In 1938 Carver donated his life's savings of over $30,000 to a foundation created by him and willed the rest of his estate to it so that his work might be carried on after his death

An incident from that book has remained in my memory with startling clarity. Carver's publications on the uses of the peanut and the sweet potato brought him to the attention of the bigwigs in Washington D.C. He was invited to the Capitol to address an eminent audience on the subject. The persons deputed to receive him at Union Station looked for a sophisticated scientist on the train arriving from Alabama and ignored the humble individual in simple working clothes who emerged carrying a battered handbag. Carver made his way to the venue for his talk. As he introduced himself to the officials, there was astonishment at the fact that the person they had dismissed was, in fact, the eminent scientist himself. Carver once explained that it was essential to avoid what he called “the ‘I’ disease.”

## My Teachers In Medical College, And Shubha

I had applied for admission to the Seth G. S. Medical College and to The Grant Medical College, two premier medical colleges in Mumbai. The letter of acceptance from the latter reached me earlier and I had already paid my fees by the time the letter of acceptance from the Seth G. S. Medical College arrived. Since I could not afford to lose the fees paid, I continued at the Grant Medical College. This was a stroke of great good fortune.

It was here that I met Shubha, later to grant me the privilege of being her husband. I have learnt much from her. She had come under the spell of Albert Schweitzer at an early age and lent me books on and by him. She decided, as an undergraduate student, to study leprosy and help patients stricken by this disease. Towards this end she accepted Dr. Noshir H. Antia's invitation to join his team at the J. J. Hospital and, on his recommendation, proceeded abroad to learn clinical electromyography so that it could be used for the early diagnosis of leprosy. (Dr. Noshir Antia was quick to see the clinical application of this new modality of testing the function of nerves and set up the first electromyography department in India. He also deserves the credit for enabling leprosy patients in Bombay Presidency to obtain inpatient treatment in general hospitals and set up the first centre in the Presidency for surgical correction of their deformities and their rehabilitation.)

Shubha learnt that Group Captain Leonard Cheshire was trying to establish a centre in Bombay for the care of incurably ill persons. She joined him in East Andheri during the weekends and assisted him in setting up a little hut and looking after his patients. Once the Cheshire home developed and no longer needed her, she joined *Snehasadan* and helped as the medical attendant in their little dispensary for slum-dwellers. She spent her weekends at this dispensary for several years. She has also helped with research at the Acworth Leprosy Hospital and at the German Leprosy Mission (later to become the Bombay Leprosy Project). Today, she is helping found a museum on the history of leprosy at the Acworth Hospital.

My teachers included Dr. B. B. Sethna, professor of Anatomy; Dr. H. S. Mehta, professor of Medical Jurisprudence and Forensic Medicine; Dr. M. M. Wagle, professor of Paediatrics; Dr. C. G. Saraiya, professor of Obstetrics and Gynaecology; Drs. Noshir Wadia, Gajendra Sinh and Vijay Dave in the Neurosciences; Dr. Noshir Antia, professor of Plastic and Reconstructive Surgery; Dr. W. D. Sulakhe, professor of Medicine; Dr. Darab K. Dastur, professor of Neuropathology; Dr. Fazl F. Chhatriwala and others who moulded my character. I shall restrict myself to some of the lessons learnt from just two of them.

Dr. Vijay Dave was an honorary consultant in Neurosurgery when I was an intern and resident doctor and yet, during a ward round, he would sincerely and earnestly seek the opinions of students and residents on patients posing clinical problems. He considered our suggestions gravely and commented on their merits. If he found them noteworthy, he recorded them on the case paper and implemented them in the management of the patient. This was in sharp contrast to many other consultants who considered themselves founts of wisdom and experience and spouted aphorisms and diktats, never pausing to listen to students or residents. Dr. Dave also taught us that at times it was necessary to doubt our competence and search for solutions, especially when the illness was grave and the problems posed by it complex.

Dr. Darab Dastur spent hours studying the tissues from a single patient, be they from the operation theatre or the autopsy room. When a resident doctor showed the slightest evidence of curiosity or the inclination to learn, Dr. Dastur would make him sit by his side after office hours, and go over slide after slide, pointing out minute details. The shape and size of the cell; the nucleus and its characteristics; the cytoplasm and its organelles; the supporting tissue; the endothelium, media and adventitia of blood vessels in and around the diseased area; the products of disease such as caseous tissue; abnormal cells, be they inflammatory or neoplastic – all these and more were brought into sharp focus. How could any student forget the havoc played by the *Mycobacterium Tuberculosis* when he was shown the findings under the microscope accompanied by such exclamations as, ‘This is mass murder of neurons!’ It did not matter to Dr. Dastur that dusk was long past and that the night-lights had come up outside the research building. He would pause only when he had completed his demonstration and dictated a detailed histopathology report. I do not see the likes of his reports these days.

## Dr. Homi M. Dastur

I completed my training as a neurosurgeon at the Grant Medical College and J. J Hospital. Disinclination for private practice prompted me to join Dr. H. M. Dastur's department at the Seth G. S. Medical College and K. E. M. Hospital. The full-time teaching position suited me admirably and in Dr. Dastur I had a mentor *par excellence*. Let me provide a few examples of what I learnt from him.

He commanded respect by his excellence in Neurosurgery and Neuroradiology, as also his integrity and sterling qualities. He was especially concerned about poor patients and did his best to provide care of the highest standard for them. In order to ensure that no patient was ever asked to leave without care, he set up a daily Neurosurgery outpatient clinic. This was unprecedented but he overcame resistance by pointing out that it was unfair to ask patients with grave illnesses who had travelled long distances to go away untreated and return on a single, specified, outpatient day.

Dr. Dastur strode into the hospital promptly on time and left only after all the work had been attended to and he had put in a minimum of eight hours of work. He built the department of Neurosurgery from nothing to one that was nationally respected. He set up a system of patient records that enabled us to pull out the case paper, x-ray films, pathology report and findings on follow-up evaluation within minutes of the patient presenting at the outpatient clinic, even after a lapse of decades. Lacking his own Neuroradiologist, Dr. Dastur developed the section of Neuroradiology to a level of excellence where Dr. Jamshed Sidhva, the honorary Neuroradiologist at the J. J. Hospital, would consult him on patients posing neuroradiological dilemmas. He developed embolisation of carotid artery – cavernous sinus fistulae, selective catheter angiography of each intracranial arterial trunk and even spinal angiography. He published papers that remain classics, especially in the field of tuberculous disease of the nervous system and craniovertebral anomalies.

These achievements, by themselves, would have evoked admiration but there were other qualities that set him apart.

When Cupid's arrow struck him and Dr. Dastur wedded his bride, the event was marked by a day-long absence. He was back at work the next day and only those very close to him knew of the reason for that absence.

He was gentle and soft-spoken but did not brook political or administrative interference. A powerful municipal councillor or a minister in the state cabinet was treated as was any other patient, no preference being offered. The individual throwing his weight around was asked to seek treatment elsewhere since the conditions at this department were not to his liking. Courtesy was inextricably blended with discipline.

We had occasion to treat an ageing Parsi gentleman for spinal disease. He was relieved of his symptoms. After he was discharged from hospital, this gentleman visited Dr. Dastur's residence during his absence and left a box of sweets. On his return Dr. Dastur found the box heavier than expected and on opening it found bars of silver beneath the layer of sweets. The next morning he attended the outpatient service and then applied for leave for a few hours. He looked up the gentleman's address in our case records and went to his home. Politely but firmly, he explained that he could not accept this generous gift, as hospital rules did not permit it. The patient graciously accepted the explanation and silver bars. If he was surprised at this unusual event, he did not display it.

## Professor Valentine Logue

Dr. Dastur made it possible for me to spend a year with Professor Valentine Logue at the Institute of Neurology (as it was known then) at Queen Square, London. Professor Logue was the doyen of Neurosurgery at this Mecca of the Neurosciences and was reputed to be the Neurologist's Neurosurgeon. The Neurophysicians at the Institute – justly acclaimed masters in their own fields – often sought Professor Logue's opinion and advice on tricky clinical problems.

I was fascinated by the fact that Professor Logue was to be found in the Institute wards on all Sundays and holidays. On enquiry, I learnt from him that he preferred examining patients with complex illnesses on holidays as he could do so without fear of interruption or the need to rush to the clinic or the operation theatre. Patiently, he heard the narration of the evolution of symptoms from the ill individual and family members. A thorough examination followed. After he had studied the findings of tests performed thus far, he collected his thoughts, analysed the diagnosis and proposed treatment for the benefit of the residents accompanying him and then talked to patient and family. He used simple terms and took pains to explain concepts that may cause difficulties. A simple example will suffice here. Instead of saying that the rate of complications from a given operation was ten percent, he would say, ‘If this operation is performed on ten patients with your illness, one of them may develop the following complications.’ I have tried to emulate the manner in which he explained the nature of the illness, the necessary tests and treatment and the likely outcome when talking to my own patients.

## Learning From Biographies And Other Published Works

During my enforced sabbatical in 1956, my uncle, Jitendrakaka, introduced me to Ashokbhai Vaidya. Ashokbhai was a senior medical student at Seth G. S. Medical College. He, in turn, introduced me to Sir William Osler and his book entitled *Aequanimitas and other addresses*. Ashokbhai counselled a study of the history of medicine. As I followed his advice, I found one work leading to another. Ashokbhai has set in motion a life-long pursuit of knowledge of the origins of our profession and the lives of those who have contributed to the development of the art and science of medicine. Better still, medicine has led me to literature, philosophy, art, natural history and much, much more. I am on the trail of unending treasures!

Sir William Osler, in turn, introduced me to his bedside library. As you may recall, this consisted of the following works:

Old and New Testaments

The Works of Shakespeare

Montaigne's *Essays*

Plutarch's *Lives*

Marcus Aurelius′ *The meditations*

Sir Thomas Browne's *Religio Medici*

Epictetus′ *The Enchiridion*

Miguel de Cervantes′ *Don Quixote*

Emerson's *Works*

Oliver Wendell Holmes — *Breakfast-Table Series*

Let me take just one item from this list. Fortunately for me, the volumes comprising Dr. Oliver Wendell Holmes′ *Breakfast-Table Series* were available at the Petit Library in Bombay and were eagerly devoured. (They are now available on the Internet and can be downloaded from the Project Gutenberg site. *The Poet at the Breakfast Table,* for example, is available at http://www.worldebooklibrary.com/eBooks/Gutenberg/etext01/ ptabt11.htm)

It was a short step from this series by Dr. Holmes to biographical notes on him, the discovery of the fact that he coined the word ‘anaesthesia’, and of his work on puerperal fever. The works of his equally eminent son, Justice Oliver Wendell Holmes, and of the doctor's friends and colleagues in Boston and elsewhere, led to different trails.

## Dr. Albert Schweitzer

As I close this essay, I return you to Dr. Schweitzer, whose life and work are sources of deep inspiration. Schweitzer could have embarked on a career in classical music with his acquired expertise on the work of Johann Sebastian Bach. He could have been a theologian. His book entitled *The quest of the historical Jesus* (1906) and other volumes such as *The mysticism of Paul the Apostle* (1930) testify to his eminence in this field. He could have been a philosopher and ethicist. Many consider his concept of *Reverence for life* to be his greatest single contribution to humankind. His philosophy has been compared with that of St. Francis of Assisi. He chose to remain a physician. He spent most of his life in Lambarene, Gabon, in west-central Africa. He set up a hospital near an already existing mission post, treated and operated on thousands of poor patients. He took care of a large number of patients with leprosy and treated many victims of the African sleeping sickness. In 1914, as Germans on French territory, Schweitzer and his wife were taken captive and interned in France. In 1924 he returned to Lambarene, where he rebuild his decayed hospital and resumed medical practice. From 1952 until his death he also joined Albert Einstein and Bertrand Russell to protest against nuclear tests and bombs.

For me, his defining teaching is that of the fellowship of those who bear the mark of pain. I leave you to ponder two quotations on this subject:

‘Pain is a more terrible lord of mankind than even death himself.’ (On the Edge of the Primeval Forest, p. 92.)

′The Fellowship of those who bear the Mark of Pain. Who are the members of this fellowship? Those who have learned by experience what physical pain and bodily anguish mean, belong together all the world over; they are united by a secret bond. One and all they know the horrors of suffering to which man can be exposed, and one and all they know the longing to be free from pain. He who has been delivered from pain must not think he is now free again, and at liberty to take life up just as it was before, entirely forgetful of the past. He is now a ‘man whose eyes are open’ with regard to pain and anguish, and he must help to overcome those two enemies (so far as human power can control them) and to bring to others the deliverance which he has himself enjoyed. The man who, with a doctor's help, has been pulled through a severe illness, must aid in providing a helper such as he had himself, for those who otherwise could not have one. He who has been saved by an operation from death or torturing pain, must do his part to make it possible for the kindly anaesthetic and the helpful knife to begin their work, where death and torturing pain still rule unhindered. The mother who owes it to medical aid that her child still belongs to her, and not to the cold earth, must help, so that the poor mother who has never seen a doctor may be spared what she has been spared. Where a man's death agony might have been terrible, but could fortunately be made tolerable by a doctor's skill, those who stood around his deathbed must help, that others, too, may enjoy that same consolation when they lose their dear ones.

‘Such is the Fellowship of those who bear the Mark of Pain.’ (On the Edge of the Primeval Forest, pp. 173.)

## Concluding Remarks

I have been blessed more than I deserve.

I have learned much from my parents (especially my mother), my wife, my teachers in school and college, the chiefs of clinical departments in which I served as resident doctor, Dr. Homi Dastur and my colleagues past and present. Most of all, I have learned from my patients, especially the poor patients who sought our help at the J. J. and K. E. M. Hospitals. The gratitude they showed and affection they showered were humbling.

As a child, I hankered after books, seeking them everywhere and seizing every opportunity at reading them. We now have a modest library of our own where we can learn from our chosen masters at will.

I have tried in a small way to pass on my blessings to younger colleagues and inspire them, as I was inspired. I hope that they will continue to help and inspire their juniors. In doing so, they will continue our ancient tradition of guru-shishya parampara*

## Questions That This Paper Raises

1. Are there, truly, turning points in our development where one or more striking experiences change our lives forever? Can you recall such moments in your own evolution?
2. Is the author trying to be an idealist? Is it not necessary to be practical in this world? It is all very well preaching morality and goodness and the interests of the poor but surely one has to survive, cater to the growth and development of one's own family and children. What is wrong in aspiring for riches - a mansion, Mercedes and other cars and annual holidays in countries such as Switzerland?
3. Can books ever really serve to inspire? Can you think of any books that have inspired you?
4. The author never refers to religion. Is he not erring gravely?

## About the Author

***Dr. Sunilkumar Krishnalal Pandya,** eminent Neurosurgeon and thinker on medical ethics, joined the Grant Medical College in 1957 and trained at the Sir J.J. Group of Hospitals, Mumbai. He obtained the M.B.B.S. (1961) and M.S. (1965). He joined Dr. Homi Dastur at the Department of Neurosurgery, Seth G. S. Medical College and K. E. M. Hospital in 1967 as a Pool Officer and was appointed to the staff as Assistant Neurosurgeon in 1968. In 1975, on Dr. Dastur's retirement, he was appointed Professor of Neurosurgery. He retired on superannuating in 1998 and has, since, worked at the Jaslok Hospital and Research Centre, Mumbai. He is Editor Emeritus, IJME (Indian Journal of Medical Ethics); Journal Ombudsman,* JPGM (Journal of Postgraduate Medicine); and *Member, International Editorial Advisory Board of the Mens Sana Monographs.*

## Footnotes

* Having a lakh (100 thousand of ruppees; in other words, a wealthy person

* The hallowed tradition of the teacher (guru) and taught (shishya) in India.

